# Attacks to Automatous Vehicles: A Deep Learning Algorithm for Cybersecurity

**DOI:** 10.3390/s22010360

**Published:** 2022-01-04

**Authors:** Theyazn H. H. Aldhyani, Hasan Alkahtani

**Affiliations:** 1 Applied College in Abqaiq, King Faisal University, P.O. Box 400, Al-Ahsa 31982, Saudi Arabia; 2 College of Computer Science and Information Technology, King Faisal University, P.O. Box 400, Al-Ahsa 31982, Saudi Arabia; hsalkahtani@kfu.edu.sa

**Keywords:** in-vehicle network, CAN, cybersecurity, intrusion detection, deep learning, artificial intelligence

## Abstract

Rapid technological development has changed drastically the automotive industry. Network communication has improved, helping the vehicles transition from completely machine- to software-controlled technologies. The autonomous vehicle network is controlled by the controller area network (CAN) bus protocol. Nevertheless, the autonomous vehicle network still has issues and weaknesses concerning cybersecurity due to the complexity of data and traffic behaviors that benefit the unauthorized intrusion to a CAN bus and several types of attacks. Therefore, developing systems to rapidly detect message attacks in CAN is one of the biggest challenges. This study presents a high-performance system with an artificial intelligence approach that protects the vehicle network from cyber threats. The system secures the autonomous vehicle from intrusions by using deep learning approaches. The proposed security system was verified by using a real automatic vehicle network dataset, including spoofing, flood, replaying attacks, and benign packets. Preprocessing was applied to convert the categorical data into numerical. This dataset was processed by using the convolution neural network (CNN) and a hybrid network combining CNN and long short-term memory (CNN-LSTM) models to identify attack messages. The results revealed that the model achieved high performance, as evaluated by the metrics of precision, recall, F1 score, and accuracy. The proposed system achieved high accuracy (97.30%). Along with the empirical demonstration, the proposed system enhanced the detection and classification accuracy compared with the existing systems and was proven to have superior performance for real-time CAN bus security.

## 1. Introduction

The technology of self-driving vehicles and smart cars has been notably improved during recent years. The term vehicular networks refers to vehicle nodes that offer advantages such as managing traffic, parking, and avoiding accidents [1]. Vehicle nodes function as a communication messenger and are studied in different research areas, for example, vehicular ad hoc networks, the Internet of vehicles, and vehicle-to-everything communications. An independent area of research, the in-vehicle networks (IVNs), deals with the communication between the engine control unit (ECU), the transmission control unit, the anti-lock braking system, the body control modules, and various sensors inside the vehicle [2].

**Figure 1:**
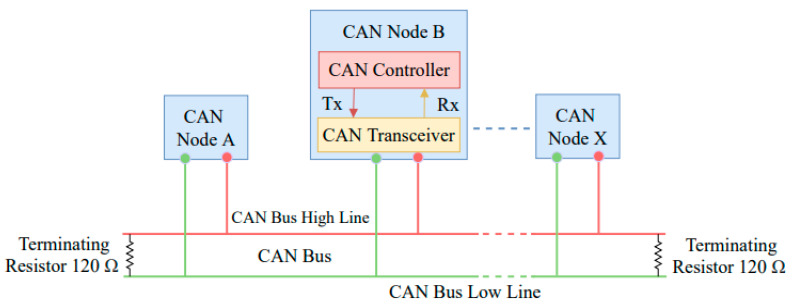
The CAN bus interface.

There are special protocols that facilitate the functioning of IVNs. These protocols include the controller area network (CAN), FlexRay, and Ethernet [3]. CAN is the most common network topology used for controlling the automotive and the industrial system. It is a communication network that offers rapid communication among microcontroller devices. CAN employs interconnected nodes to send a message-based protocol designed to permit all nodes to receive the message and perform on the network message [4]. Figure 1 shows the CAN standard bus interface that attackers use to inject attack messages into the communication network.

**Figure 2:**
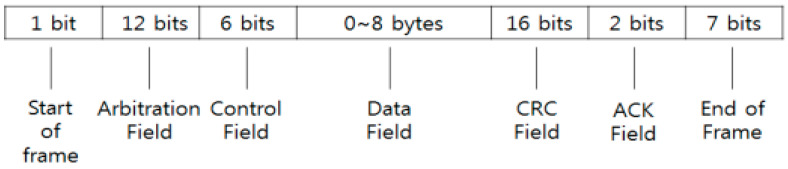
CAN bus data Frame.

Figure 2 shows the CAN message header frame format which consists of the start of the frame (1 bit), in the arbitration field (12 bits); the arbitration field is used to determine the owner of the CAN message when the system starts broadcasting. The cyclical redundancy check (CRC) was used to check the frame header and uses the (16 bits), Acknowledge (ACK) field to return messages to the network for receiving the frame; the end of frame (EOF) has (7 bits). 

Two important inventions are emerging as ways to offer drivers more convenience: high connectivity and automotive electronics [5]. Vehicle-to-vehicle communication uses smart devices and the cellular network to allow drivers to share important information such as dangerous situations on the road. Another type of communication is vehicle-to-infrastructure, which is incorporated in autonomous vehicles in the form of sensors. The novel developments in technology have made vehicle smart devices that are equipped with specific instruments that offer safety (e.g., forward collision avoidance) and convenience (e.g., telematics) [6,7]. However, these improvements in vehicle connectivity are prone to external attacks. For example, the current CAN message frame does not have authentication mechanisms, leading to the lack of security for the in-vehicle data [8]. In addition, the interconnection of in-vehicle controllers is accompanied through an increase in the complexity of the architecture. Thus, unintended motions or failures can be caused by mutual effects between controllers, which may lead to defects affecting the safety of the passengers or the cybersecurity of the vehicles [9,10,11].

Certain procedures must be considered when designing the cybersecurity of a mission-critical environment such as vehicles. IVNs protection requires intrusion detection or prevention systems of high accuracy [12]. A vehicle may recognize a critical message as an attack, causing safety issues. Consequently, the intrusion prevention system should be able to block false alarms [13,14]. Malicious attacks on vehicles could pose safety problems to passengers, pedestrians, and other vehicles. Hence, real-time response is vital for the cybersecurity of vehicles. Nevertheless, the in-vehicle system does not respond in real time due to constraints in the time and space resources of the moving vehicle. This leads to the necessity of designing a real-time intrusion detection system (IDS) of high accuracy that performs within the available limited resources [15].

The CAN bus system has been shown to have technical defects, as the receiving nodes do not authenticate if a received packet whose source is not given is authorized or not [16]. Hackers can use ECUs to send unauthenticated CAN packets. Such defects make CAN bus systems vulnerable and unable to recognize the nodes responsible for the attacks. Thus, security systems for the CAN bus are important [17].

However, many challenges arise in network-based attacks since they are new to the automotive field of research [18]. Because there is an opportunity to modify the CAN protocol, a machine learning approach can be employed to apply an intrusion detection method, owing to the ability to learn through examples to adjust to any modification in the protocol. Many studies have adopted machine learning-dependent IDS that requires supervision when deployed. Data used in such studies need to be thoroughly labeled, which is impractical given the large amount of data per milliseconds produced by real-time CAN [19,20]. Consequently, a detecting system based on an unsupervised machine learning approach is needed.

In the USA, Google started examining driverless vehicles in 2009 with road tests of CAVs [21]. Tesla [22] has designed on-road CAV driving vehicles and distributed them for commercial purposes; for instance, the University of Michigan [23] has tested the in Mcity field. In Europe, major companies such as BMW, Audi, and Mercedes Benz have begun to develop CAN systems [24]. In China, the CAN system was tested in Shanghai [25], while Baidu started designing the Apollo CAV framework in 2019 [26]. Some studies have attempted to discuss intrusion in CAVs. It was indicated that spoofing and flood attacks, two of the serious cyberattacks, send fake messages [27]. The cyberattacks in CAVs have been categorized into passive and active attacks. 

**Figure 3:**
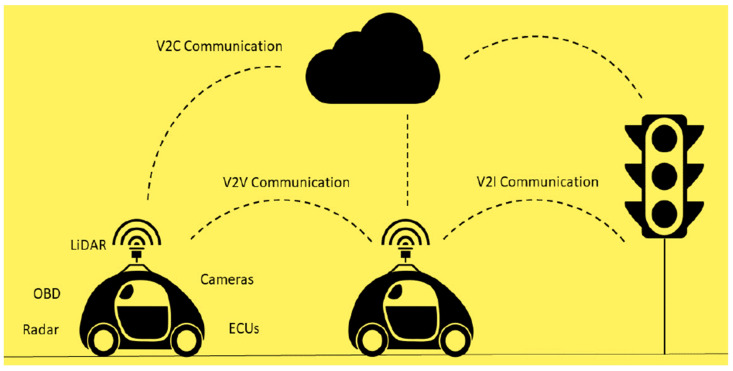
Attack points through communication.

Login password, knowledge-acquiring attacks are sorts of attacks on interconnected-computers networks [28]. Various sources of attack in traditional automobile vehicles have indeed been classified into two sorts [29], including cyberattacks on the sound system or mobile apps and attacks on the CAN. The latter sort of attack is deemed riskier than the first because CAN is interconnected to in-vehicle hardware pieces of equipment such as brakes, air conditioning systems, and the steering wheel. CAVs are integrated with both hardware and virtual software components interconnected to the complete transportation infrastructure, unlike computer networks and ordinary autos. As a result, any form of attack on a vehicle could occur in CAVs. Furthermore, as autonomy and connectivity grow, more vulnerability and attack points will occur [30]. Cybersecurity is required to secure the system against cyberattacks that could impact its effectiveness, whether electronically or physically. Utilizing the artificial intelligence model-based CAV architecture described in Figure 3, it is vital to detect, identify, and categorize different types of attacks on CAVs at an initial stage.

## 2. Related Works

The most recent research works on the intrusion detection systems on CAN are discussed in this section. Song et al. [31] used an inception-ResNet model to train the in-vehicle network traffic data against attacks to detect intrusion. The results have been compared with various existing models such as long short-term memory, the neural network (NN), the support vector machine (SVM) approach, the naïve Bayes approach, the k-nearest neighbors (KNN) model [32], and decision tree algorithms [33]. Zhang et al. [34] developed an intrusion detection system to manage the CAN bus from attacks, and the authors used a hybrid model, namely gradient descent momentum and adaptive gain, for classification of the attacks’ message. Liang et al. [35] applied deep neural network-based intrusion detection for monitoring the CAN bus message frame. For training process, the deep learning model used was the deep-belief network function, of which the accuracy of the proposed system has been shown to reach 98%. Hoppe et al. [36] developed an IDS system in the CAN bus to analyze network traffic for finding new network packets’ pattern and compared them with patterns on the IDS system. The system was compared with the tradition system, and it is noted that their system achieved high accuracy. Taylor et al. [37] introduced an LSTM model to detect CAN bus attacks. Wang et al. [38] presented a hierarchical temporal memory algorithm to design a distributed anomaly classification. The empirical results have indicated that the model requires more time to detect attacks. Several machine learning (ML) and deep learning (DL) algorithms have been applied to predict intrusions on the CAN bus, using the deep neural network [39,40], applied Convolutional Neural Networks (CNNs) [41], and artificial neural networks (ANNs) to build the adversarial attacks [42]. 

To raise awareness about the cybersecurity of vehicles, a Jeep Cherokee was remotely hacked in 2015 [43]. A recent study [44] concluded that the main focus of research should not be on preventing attacks, since it is impossible to produce a vehicle with a security system that defends it against attacks. On the contrary, attention should be paid toward designing a system that detects attacks and responds accordingly. 

Thus, the current study proposes a model that detects attacks and abnormal behaviors resulting from injected messages onto vehicles in real time with appropriate accuracy. A technique known as hierarchical data analysis was applied to detect and classify the attack data. Moreover, a machine learning algorithm was used for minimizing misdetection and non-detection by properly training the model of intrusion detection. To obtain the required hyper parameters, we provided a simulation environment and used an algorithm that is suitable for the selected dataset. More specifically, a method that promptly detects an existing attack in real time was suggested [45,46,47]. This was achieved through the CAN data behavior. To validate the model for vehicles in a real environment, we increased its accuracy and ensured its function with limited resources. To measure the accuracy of the model, the F1 score and the detection time were used as reliable metrics. The empirical results of our study showed optimal accuracy with deep learning approaches compared with other state-of-the-art approaches for detecting attack messages from a CAN bus [48].

## 3. Contribution

The main motivation for the proposed system is to address the challenges of information security in CAVs by detecting the potential attack messages and launching CAV cybersecurity. The artificial intelligence framework is one solution to the robust building for the confrontation of cyber threats to IVNs’ communication. Novel intrusion detection from IVNs’ compunction is important, considering that CAVs have become an emerging technology in many countries and are incorporated in daily social life. The development of the proposed deep learning approaches to detect attacks against in-vehicle CAN buses was the main objective of the study. This method greatly improved the detection accuracy of all types of attacks compared with the existing systems. The proposed system achieved superior accuracy in detecting two types of attacks. Furthermore, the deep learning approach detected attack messages in a CAN bus. The proposed system was examined by using recent real datasets for CAV cybersecurity. 

## 4. Materials and Methods

**Figure 4:**
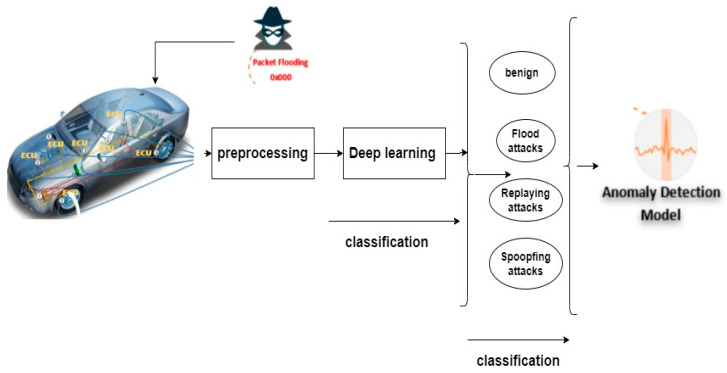
The proposed framework.

As self-driving vehicles were rapidly developed, many companies have faced challenges related to the protection of the CAV system against attacks, creating various issues on the road. A few studies have discussed approaches to secure the systems, but there is still a gap in the algorithm to obtain high performance. In this study, we used deep learning approaches on real CAV datasets. Figure 4 shows the proposed framework to detect attacks against a CAV network. 

### 4.1. Dataset

The CAV dataset was collected from real CAN traffic data including spoofing, flood and replaying attacks, and benign packets. The dataset was designed by building a CAN traffic OBD-II port from a real CAV where the transferring messages injected various types of attack messages. The CAN packet generator Open Car Testbed and Network Experiments (OCTANE) was used. The intrusions were injected every 3 to 5 sec, and CAV traffic took 30 to 40 min. Table 1 shows the injection attack of CAN traffic. Dataset available via this link https://ocslab.hksecurity.net/Datasets/CAN-intrusion-dataset (access on 20 November 2021).

### 4.2. Preprocessing

The dataset contained the information of the timestamp in seconds, data and arbitration ID features in hexadecimal and DLC, and data bytes from 0 to 8 (Table 2). The labels of the dataset received three attacks, namely spoofing, flood, and replaying attacks, as well as benign and normal packets (Table 3). To run the system, the data and arbitration ID feature are categorical variables, including the messages sent from the ECU devices to CAN. Therefore, we converted these variables to numerical to identify and classify the intrusion.

After transforming the categorical variables, the data were processed by using maximum–minimum normalization methods to avoid a possible overlap in the training process that can result from handling large datasets. In the normalization method used to scale the dataset in the same range, we used a scaling range between 0 and 1.

(1) $$z_{n}=\frac{x-x_{min}}{x_{max-x_{min}}}\left(New_{max_{x}}-New_{min_{x}}\right)+New_{min_{x}}$$

where,

$x_{min}$: minimum of the data

$x_{max}$: maximum of the data

$New_{min_{x}}$: the minimum number (0)

$New_{max_{x}}$: the maximum number (1).

### 4.3. Proposed System of the Deep Learning Algorithm

**Figure 5:**
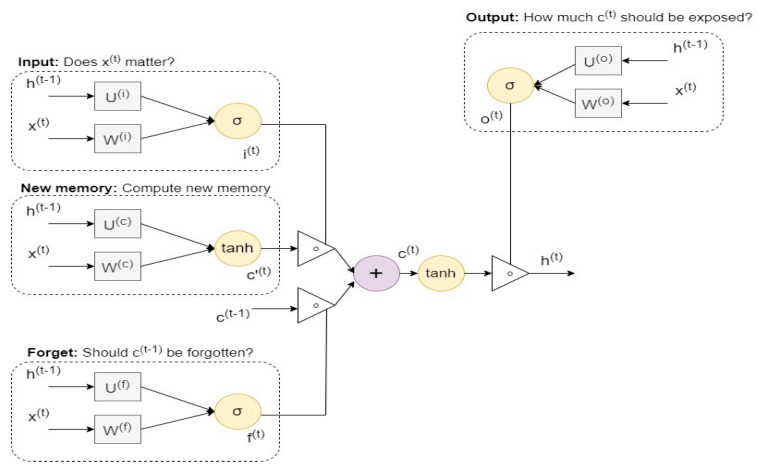
The structure of the LSTM technique.

In this study, we applied deep learning approaches to detect CAN attacks, [49] presenting the LSTM technique as a time recurrent neural network (RNN) for long-term knowledge dependency. The flow of LSTM is comparable to that of RNN. The difference between the LSTM and RNN techniques is in the way that cells operate in the case of LSTM [50]. Each LSTM unit consists of four gates: input, candidate, forget, and output. The forget gate classifies data as to whether they should be discarded or saved. The input gate refreshes the cells, and the hidden state in the LSTM is always determined by the output gate. In addition, LSTM incorporates an embedded memory block and gate structure that allow it to solve both the disappearing and the implosion-gradient difficulties in the RNN learning process [51]. The structure of the LSTM technique can be seen in Figure 5.

The computing equations that are associated with the LSTM structure in Figure 5 are as follows:

(2) $$f_{t}=\sigma \left(W_{f\;}.\;X_{t}+W_{f}.\;h_{t-1}+b_{f}\right)$$

(3) $$i_{t}=\sigma \left(W_{i}.\;X_{t}+W_{i\;}.\;h_{t-1}+b_{i}\right)$$

(4) $$S_{t}=tanh\left(W_{c}.\;X_{t}+W_{c\;}.\;h_{t-1}+b_{c}\right)$$

(5) $$C_{t}=i_{t}*S_{t}+f_{t}*S_{t-1}$$

(6) $$o_{t}=\sigma \left(W_{o}+X_{t}+W_{o}\;.\;h_{t-1}+\;V_{o}\;.C_{t}+b_{o}\;\right)$$

(7) $$h_{t}=o_{t}+\tan h\left(C_{t}\right)$$

The arithmetical notations in the above formulas can be represented as follows:

$X_{t}$ is the vector of the input data that are forwarded to the memory cell at time *t*;

$W_{i}$, $W_{f}$, $W_{c}$, $W_{o}$, and $V_{o}$ refer to the weight matrices;

$b_{i}$, $b_{f}$, $b_{c}$, and $b_{o}$ are point to bias vectors;

$h_{t}$ indicates the specified value of the memory cell at time *t*;

$S_{t}$ and $C_{t}$ are defined values of the candidate state of the memory cell and the state of the memory cell at time *t*, respectively;

σ and tanh represent the activation functions in the LSTM neural network;

$i_{t}$, $f_{t},$ and $o_{t}$ are obtained values for the input gate, the forget gate, and the output gate at time *t*, respectively. These gates have values in the range of 0 to 1 over the nonlinear sigmoid activation function.

**Figure 6:**
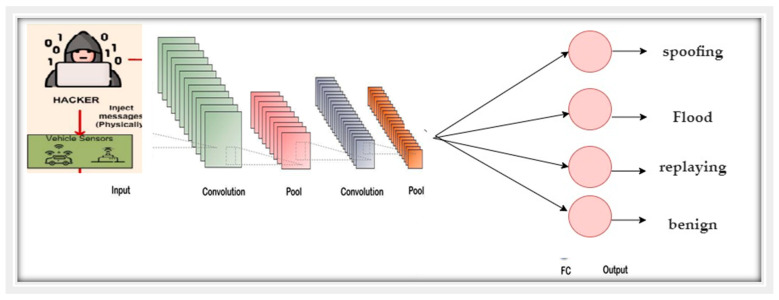
The structure of the CNN model.

CNN is one technique of the deep-learning neural network that takes spatial inputs into account. CNN neurons, as with other neural networks, possess trainable weights and biases. Furthermore, CNN is mostly employed to manage information with a grid layout, which distinguishes it from other architectures [52]. CNN is a feed-forward network with the input dataflow in one direction, from input to output [53]. The CNN model is mainly comprised of three layers: the convolutional, pooling, and fully connected layers. To reduce data dimensionality and computation cost, the convolution and pooling layers are utilized. The completely connected layer, on the other hand, is the folded layer connected to the output of the previous layers. There are different pooling techniques in the structure of CNN such as maximum, average, and global pooling. From those, maximum pooling is widely used and functions by selecting the maximum value from a pooling window. Figure 6 shows the structure of the CNN model.

**Figure 7:**
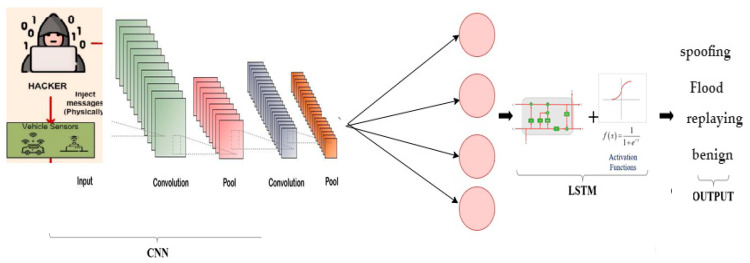
The structure of the CNN-LSTM mode.

CNN-LSTM is an integrated deep-learning algorithm based on neural networks techniques. It was created to solve problems of visual time-series forecasting and to generate text from sequences of images. CNN layers are used as an extraction feature from the input data, while LSTM is combined with CNN to allow sequential prediction in the CNN-LSTM system. CNN takes information from spatial data, applies it to the LSTM structure to generate the description [54,55], and classifies the intrusion detection system. The CNN-LSTM network effectively preserves the spatiotemporal associations and continuously beats the connected LSTM (FC-LSTM) model in precipitation prediction, according to the results of the experiment. The CNN-LSTM model’s structure is depicted in Figure 7. The significant parameters of the CNN-LSTM model is presented in Table 4. Pseudocode of CNN-LSTM algorithm is presented in Algorithm 1.
**Algorithm 1. Algorithm of CNN-LSTM***Preprocessing data* *Class 4, input data 22222* *Model = Sequential()* *model. Add(Conv1D(filters = 128, kernel_size = 1, strides = 1, padding = ‘same’, input shape = (train_data_st.shape [1], 1)))* *model. Add(Conv1D(filters = 128, kernel size = 1, strides = 1, padding = ‘same’))* *model. Add(LSTM(64, activation = ‘relu’, return sequences = True))* *model. Add(LSTM(64, return sequences = True))* *model. Add(Flatten())* *model.add(Dense(128, activation = ‘relu’))* *model.add(Dense(256, activation = ‘relu’))* *Build Model* *Input = Input(shape = (train_data_st.shape[1],1))* *C = Conv1D(filters = 32, kernel_size = 1, strides = 1)(inp)* *C2 = Conv1D(filters = 32, kernel_size = 1, strides = 1, padding = ‘same’)(C)* *A1 = Activation(“relu”)(C11)* *C3 = Conv1D(filters = 32, kernel_size = 1, strides = 1, padding = ‘same’)(A11)* *S13 = Add()([C12, C])* *A1 = Activation(“relu”)(S11)* *M11 = MaxPooling1D(pool_size = 1, strides = 2)(A12)* *C3 = Conv1D(filters = 32, kernel_size = 1, strides = 1, padding = ‘same’)(M11)* *A3 = Activation(“relu”)(C21)* *C4 = Conv1D(filters = 32, kernel_size = 1, strides = 1, padding = ‘same’)(A21)* *S4 = Add()([C22, M11])* *A4 = Activation(“relu”)(S11)* *M4 = MaxPooling1D(pool_size = 1, strides = 2)(A22)* *C5 = Conv1D(filters = 32, kernel_size = 1, strides = 1, padding = ‘same’)(M21)* *A5 = Activation(“relu”)(C31)* *C6 = Conv1D(filters = 32, kernel_size = 1, strides = 1, padding = ‘same’)(A31)* *S5 = Add()([C32, M21])* *A5 = Activation(“relu”)(S31)* *M31 = MaxPooling1D(pool_size = 1, strides = 2)(A32)* *F1 = Flatten()(M31)* *D1 = Dense(32)(F1)* *A66 = Activation(“relu”)(D1)* *D22 = Dense(32)(A66)* *D33 = Dense(labels.shape[1])(D22)* *A77 = Activation(“softmax”)(D33)* *model = Model(inputs = inp, outputs = A7)* *# opotimnaztion* *Paramters patience = 3, verbose = 1, factor = 0.5, lr = 0.00001 and optimizer = rms, epochs = 10* *batch_size = 64* *For*→*rms = keras.optimizers.rms = RMSprop(learning_rate = 0.001, rho = 0.9)* *history = model.fit(x_train_cnn,y_train, batch_size = batch_size,* *steps_per_epoch = x_train.shape[0]//batch_size,* *epochs = epochs,* *validation_data = (x_validate_cnn,y_validate),* *#validation_split = 0.10,* *callbacks = [learning_rate_reduction, checkpoint]*

### 4.4. Evaluation Metrics

In order to evaluate the proposed system, the standard evaluation of accuracy, recall, precision, and F1-score metrics was applied. The evaluation metrics calculate by using confusion metrics indicators namely true-positive (TP), false-positive (FP), true-negative (TN), and false-negative (FN).

(8) $$\mathrm{Accuracy}=\frac{\mathrm{TP}+\mathrm{TN}}{\mathrm{FP}+\mathrm{FN}+\mathrm{TP}+\mathrm{TN}}\times 100\%$$

(9) $$\mathrm{Precision}=\frac{\mathrm{TP}}{\mathrm{TP}+\mathrm{FP}}\times 100\%$$

(10) $$F1-\mathrm{score}=2*\frac{\mathrm{precision}\times \mathrm{sensitivity}}{\mathrm{precision}+\mathrm{sensitivity}}\times 100\%$$

(11) $$\mathrm{Specificity}=\frac{\mathrm{TN}}{\mathrm{TN}+\mathrm{FP}}\times 100\%$$

## 5. Experiments

The CAN packets generator OCTANE was used to collect the training data for the examination of the proposed deep learning algorithm. In this experiment, we applied two deep learning algorithms, namely CNN and CNN-LSTM.

### 5.1. Splitting the Dataset

The dataset was divided into 70% of data for the training and 30% for the testing. The testing data were used to validate and evaluate our model for attack detection from the vehicle’s self-care system. Table 5 shows the splitting of the dataset.

In this experiment, the network packets were 800,860. The testing process included 240,258 packets considered as the testing data. The validation process was applied to avoid overfitting issues occurring during the training process.

### 5.2. Environment Setup

To develop the cybersecurity system by using artificial intelligence algorithms, the hardware and software parts were required to successfully obtain the system. Table 6 summarizes the system requirements for the development of the proposed security system.

### 5.3. Results

The proposed deep learning models were used to identify the attack messages from the vehicle network. The system was examined by applying a real network which included fuzzing, spoofing, replaying, and normal packets. The datasets were randomly divided into 70% of the data for training and 30% for testing. The database of the system contained 486,640 messages in the training phase and 486,640 messages in the testing phase.

**Figure 8:**
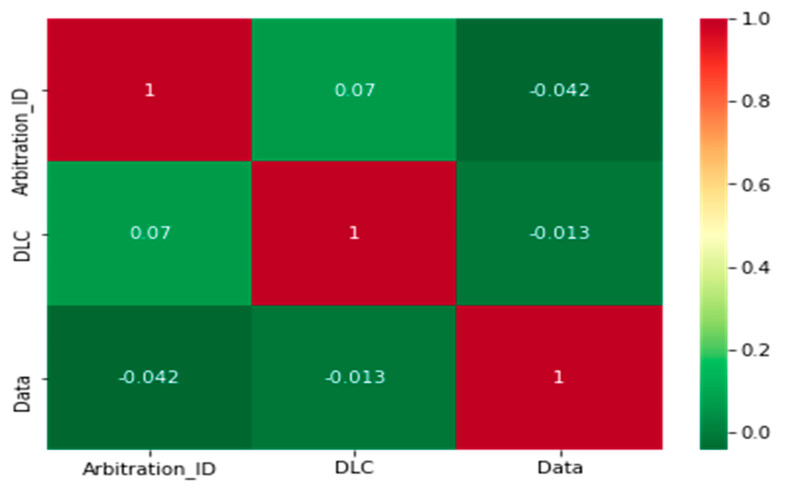
Correlation features of the dataset.

Table 7 shows the statistical analysis of the datasets, the mean, maximum, and minimum values, and the standard deviation metrics for the specific dataset features. The statistical results revealed that there is a large difference between the features and the labels. We noted that the traditional approaches used to detect the attack messages in a CAN bus are not appropriate. Figure 8 displays the correlation between the features of the datasets. There is a gap between the features due to the different characteristics of the network.

Table 8 shows the results of the CNN model for attack detection. As for the precision (0.86%), recall (100%), specificity (93%), and F1-score (100%), they achieved good values. However, the CNN model failed to detect the attack packets. Overall, the performance of the CNN model in the identification of attack messages from a CAN bus was 86%. As we mentioned earlier, the monitoring of the traffic of a CAN bus poses big challenges, therefore we developed a hybrid deep learning model that deals with these attacks.

**Figure 9:**
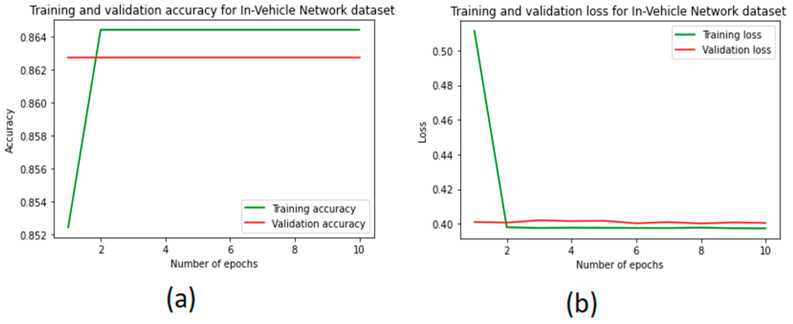
The performance of the CNN model: (**a**) accuracy performance and (**b**) training loss and validation.

Figure 9 shows the performance, the loss of training, and the validation of the CNN model to predict attacks in a vehicle network. Figure 9a shows the accuracy of the CNN model with 10 epochs. We observed that the accuracy of the CNN model increased from 84% to 86% and then reached a plateau. Therefore, we considered 10 epochs. Figure 9b shows the loss of training in the CNN model. It can be noted that the training loss decreases very slowly due to the decreased accuracy performance, starting from 0.52 and reaching 0.40.

In order to improve the training accuracy, the overfitting of the proposed system should be overcome. Therefore, the hybrid CNN-LSTM model was applied. Table 9 summarizes the CNN-LSTM results of the detection of the attack messages from a CAN bus. The proposed system failed to detect replaying and spoofing attacks. However, the CNN-LSTM model achieved superior performance in the detection of the flood, fuzzing, and normal packets. The overfitting of the system was overcome by using a hybrid deep learning approach.

**Figure 10:**
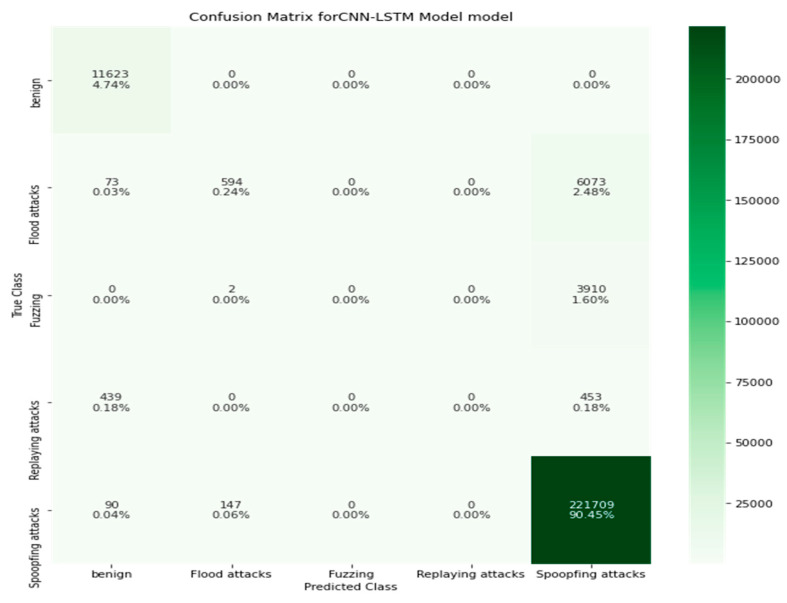
Confusion metrics of the CNN-LSTM model.

The confusion metrics, in terms of TP, FP, TN, and FN, are important in the evaluation and classification of the CAN messages in the proposed system. Furthermore, the confusion metrics calculate the number of CAN messages correctly classified as normal or attacks. The confusion metrics of the CNN-LSTM model are presented in Figure 10. Prediction values of each class is presented in percentage values.

**Figure 11:**
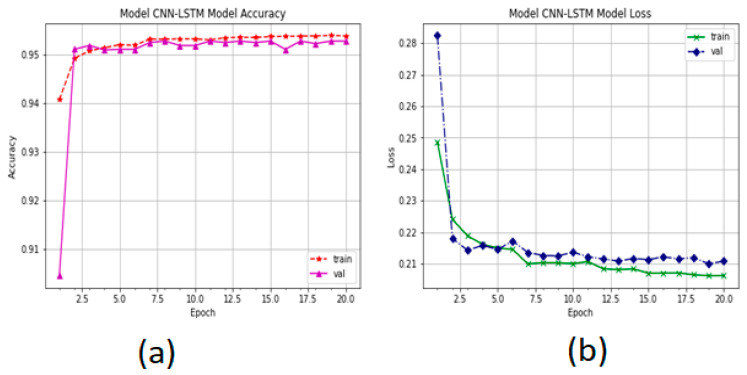
The performance of CNN-LSTM: (**a**) accuracy performance and (**b**) training loss and validation of the CNN-LSTM model.

The accuracy performance of the proposed system is presented in Figure 11. The *y*-axis represents the percentage of corrected classified. The training accuracy is the performance of the validation system. We observe that the system stopped the optimization to increase the accuracy to 20 epochs. The performance of the CNN-LSTM model increased from 91% to 95.55%. The categorical_crossentropy function was used to measure the training loss of the proposed system. Figure 11b shows the CNN-LSTM loss. It is also observed that the validation loss decreased from 24 to 20, whereas the training loss decreased from 25 to 21 with 20 epochs.

**Figure 12:**
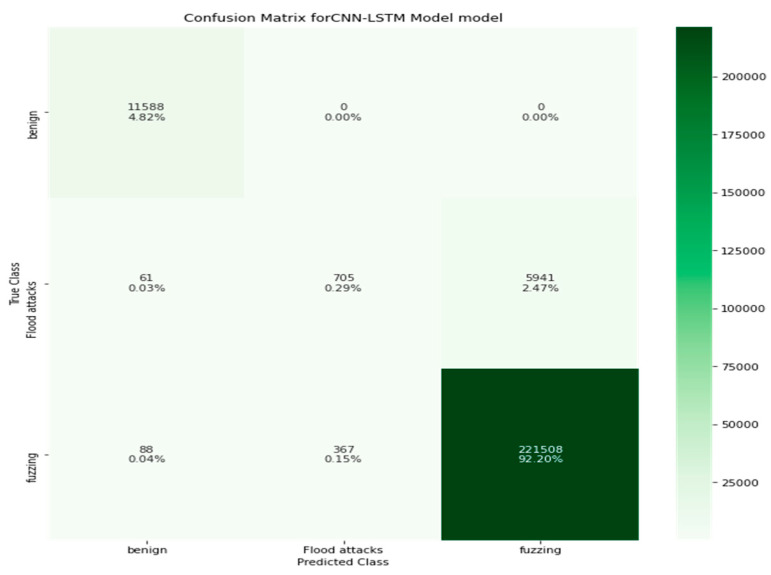
The confusion metrics of the CNN-LSTM model in the detection of the flood, fuzzing, and normal packets in a CAN bus network.

Table 10 shows the experimental results of the CNN-LSTM model in the evaluation of flood and fuzzing attacks and normal packets. It is noted that the performance of the proposed system was enhanced. The evaluation metrics of the weighted values are precision (97%), recall (97%), F1-score (96%), and accuracy (97.30%). The empirical results showed that, when the replaying and spoofing attacks were removed, the accuracy of the system increased. Figure 12 displays the confusion metrics of the CNN-LSTM model in the detection of flood and fuzzing attacks and normal packets in a CAN bus.

**Figure 13:**
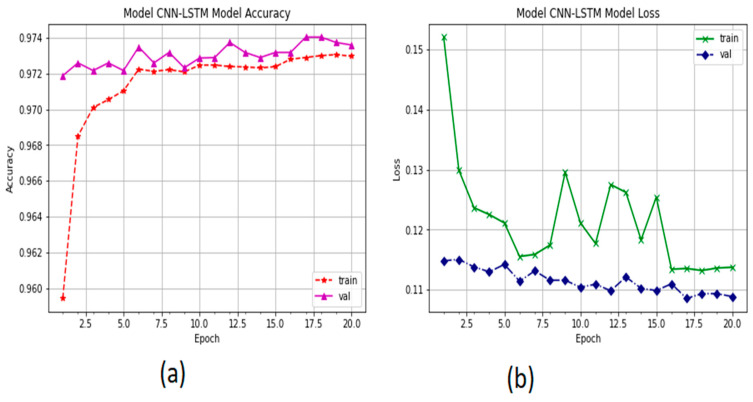
The performance of CNN-LSTM: (**a**) accuracy performance and (**b**) training and validation loss of the CNN-LSTM model in detecting flood, fuzzing, and normal packets in a CAN bus.

The validation performance of the proposed model for identifying fuzzing attacks and normal packets in a CAN bus is presented in Figure 13. The system achieved a validation accuracy of 97%, undergoing an increase from 94% to 97.74% with 20 epochs. The validation loss is minimal due to the very slight overfitting of the system, and the validation loss is reduced to 0.11 by using cross entropy metrics.

## 6. Discussion

With the increase in CAV manufacturing, companies are developing and adding new features that make care smarter. These features are connected to remote networks, therefore risk will inevitably increase. Hackers try to find a gap in the CAN bus system by sending fake messages that contain incorrect information. Intrusion detection in autonomous vehicle networks has played a significant role in the detection of malicious traffic and the monitoring of CAN bus systems for the identification of normal and abnormal messages among different ECUs. The IDS can be developed by employing artificial intelligence models such as machine learning and deep learning algorithms that handle databases containing numerous attacks and normal packets to detect new attacks.

In this study, we investigated a deep learning model that identifies attack behaviors in a CAN bus. In order to evaluate the proposed system, experimental data were used to detect attack messages in a CAN bus system. First, we applied a CNN model to predict and classify the dataset with two labels: normal or attacks. We observed that the model had more overfitting, and the accuracy was good. In the second experiment, the hybrid CNN-LSTM model was applied to identify intrusion from a dataset with four labels/types of attack, namely flood, fuzzing, spoofing, and replaying attack and a normal packet. In the third experiment, we applied the CNN-LSTM model with a dataset containing flood, spoofing, fuzzing, and normal packets. The performance of the proposed dataset was high compared with a different dataset. Table 11 shows the final results of the proposed system.

**Figure 14:**
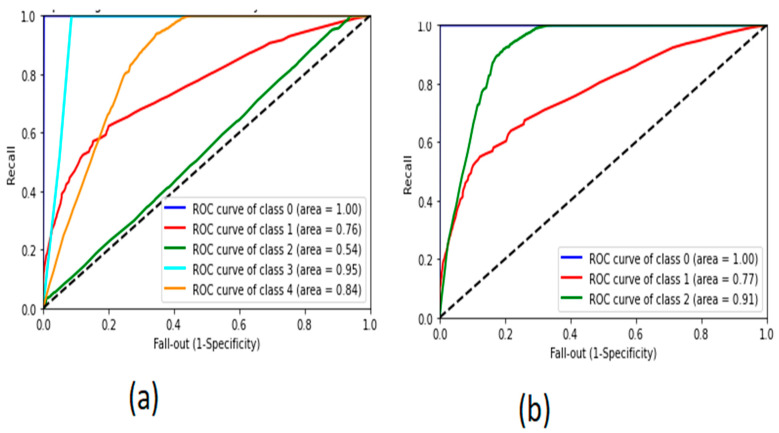
The receiver operating characteristics curve of CNN-LSTM: (**a**) dataset with two classes and (**b**) dataset with three classes.

The proposed system achieved the highest accuracy with the dataset of four classes containing flood, spoofing, fuzzing, and normal packets. The graphical representation of the receiver operating characteristic curve is shown in Figure 14, demonstrating the performance of the model in the classification of all classes.

A comparative classification performance between the proposed system and existing models is presented in Table 12. The accuracy of the proposed framework scored 97%, outperforming all the present systems for detecting IDS on vehicle networks.

## 7. Conclusions

With the rapid development of automobile manufacturing and the Internet of Things technology, the autonomous vehicle network has become intelligent and more established. The autonomous vehicle provides many facilities by connecting the automobile to satellite navigation or entertaining systems. However, autonomous cars providing these facilities face the risk of remote attacks due to the connection of the intelligent automatic vehicle network to the Internet for remote accessing.

The traffic behavior of CAN is a broadcast domain in nature. The development of an efficient security system has faced a lot of challenges. Hence, the intrusion detection system based on artificial intelligence models has given solutions against the increased risk of vehicle networks. IDS based on artificial intelligence algorithms can update the system if there are any changes in the CAN messages sent from possible attackers.

In this paper, we proposed a novel intrusion detection system for attacks against a CAN bus by using a large real dataset containing spoofing, flood, and replaying attacks, as well as benign packets. The CAN bus system was injected with various types of attack messages to generate a real dataset with different time intervals for the evaluation of the system using OCTANE.

The empirical results established that the proposed CNN-LSTM and CNN models identify attack messages. The proposed systems were confirmed to efficiently display abnormal packet detection to protect the CAN bus. They can also be extended to other designs of security systems within the complex infrastructures of autonomous vehicle networks for secure data processing.

Overall, the proposed systems achieved an accuracy score of 97.30%. These empirical results were compared with existing systems, outperforming them. In the future, we will continue improving our system by using advanced artificial intelligence.

## Figures and Tables

**Table 1:** CAN bus attacks.

Attacks	Description
Flood Attack	Sending flood messages from CAN to different ECU nodes. The attacks were injected every 0.3 ms.
Replaying Attack	The replaying attacks send a message to CAN, earlier sent by users that have injected CAN messages containing replaying attacks. The injections occurred every 0.5 ms.
Spoofing Attack (RPM/gear)	Injecting attacks to CAN messages related to RPM/gear information. They were injected every 1 ms.

**Table 2:** Features of the dataset.

Feature	
Timestamp	recorded time (s)
CAN ID	identifier of CAN message in HEX (ex. 043f)
DLC	number of data bytes, from 0 to 8
DATA [0~7]	data value (byte)

**Table 3:** Training datasets for each class.

#Labels	Volume
Flood attack	38,657
Replaying attack	13,294
Spoofing attack	2890
Normal packets	739,679
Fuzzing	22,527

**Table 4:** Parameters of the proposed model.

Parameters	Size of Values
Convolutions layer	128
Kernel size	5
Size of max pooling	5
Size of Drop out	0.50
Size of Fully connected	256
Name of Activation function	tanh
Optimizers function	RMSprop
Learning_rate	0.001

**Table 5:** Splitting the dataset.

#Data	#Instance Values
Training	490,526
Testing	240,258
Validation	70,076

**Table 6:** Hardware and software requirements for the design of the system.

Hardware	Software
8 GB RAM	Python
CPU I7	Jupyter
	Operating System: Windows

**Table 7:** Statistical analysis.

Features	Mean	Standard Deviation	Minimum	Maximum
Arbitration ID	1.80	1.67	0.00	8.00
DLC	7.50	1.188	2.00	8.00
Data	1.61	5.98	0.00	2.78

**Table 8:** Results of the proposed system for the validation phase.

Dataset	Precision (%)	Recall (%)	F1-Score (%)
Normal	0.86	100	93
Attacks	0.00	0.00	0.00
Accuracy	0.86		
Weighted average	0.75	0.86	0.80

**Table 9:** Results of the CNN-LSTM model in the detection of all attacks on the dataset of a CAN bus.

Attacks	Precision %	Recall %	F1-Score %
Benign	95	100	97
Flood	91	0.09	0.16
Replaying	0.0	0.0	0.0
Spoofing	0.0	0.0	0.0
Fuzzy	96	100	98
Accuracy 95.44%			
Weighted average	93	95	93
Loss 0.20			

**Table 10:** Results of the CNN-LSTM model for the detection of the flood, fuzzing, and normal packets in a CAN bus.

Attacks	Precision %	Recall %	F1-Score %
Benign	99	100	99
Flood	66	11	18
Fuzzy	97	100	99
Accuracy	97.30%		
Weighted average	97	97	96
Loss 0.11			

**Table 11:** Comparison results of deep learning algorithms.

Models	Labels	Precision (%)	Recall (%)	F1-Score (%)	Accuracy (%)
CNN	Two	75	86	80	86
CNN-LSTM	Six	93	95	93	95.44
CNN-LSTM	Three	97	97	96	97.30

**Table 12:** Shows accuracy performance of recent research against the proposed system on intrusion detection system for in-vehicle networks.

Ref.	Models	Accuracy %	Attack Types
Ref. [56]	Deep learning model	95%	Normal and attacks (Two classes)
Ref. [57]	Deep learning model	85%	DoS, Command Injection, Malware attacks
Ref. [58]	Generative adversarial networks	95%	DoS, Fuzzing, and Gear attacks
Ref. [59]	LSTM	80%	Spoofing, Replay, and Flooding attacks
Ref. [60]	Machine learning	90%	DoS, Fuzzing, Spoofing attacks
Ref. [61]	Neural network–LSTM	90%	DoS, Fuzzing, Spoofing attacks
Proposed model	CNN-LSTM	97%	DoS, Fuzzing, Spoofing, Replaying

## Data Availability

The data presented in this study are available here https://ocslab.hksecurity.net/Datasets/datachallenge2019/car.
